# Serotype distribution and antimicrobial resistance of invasive *Streptococcus pneumoniae* isolates among Croatian adults during a fifteen-year period (2005-2019)

**DOI:** 10.3325/cmj.2022.63.156

**Published:** 2022-04

**Authors:** Iva Butić, Marija Gužvinec, Marko Jelić, Irena Groš, Sandra Lucić, Mile Bošnjak, Arjana Tambić Andrašević, Working Group for Invasive Isolates of the Croatian Committee for Antibiotic Resistance Surveillance

**Affiliations:** 1 Department of Clinical Microbiology, University Hospital for Infectious Diseases Dr Fran Mihaljević, Zagreb, Croatia; 2 School of Dental Medicine, University of Zagreb, Croatia; 3 Faculty of Economics and Business, University of Zagreb, Zagreb, Croatia

## Abstract

**Aim:**

To assess serotype distribution, antibiotic resistance, and vaccine coverage against *Streptococcus pneumoniae* causing invasive infections in Croatian adults from 2005 to 2019.

**Methods:**

In this retrospective study, invasive pneumococcal strains were collected through a microbiological laboratory network with country coverage >95%. Capsular typing was performed with the Quellung reaction. *In vitro* susceptibility testing was carried out according to the European Committee on Antimicrobial Susceptibility Testing guidelines. In macrolide-resistant isolates, the presence of *ermB* and *mefA* genes was evaluated.

**Results:**

During the fifteen-year study period, 1123 invasive pneumococcal isolates were obtained. The most prevalent serotypes were 3, 14, 19A, 9V, 7F, and 23F, comprising 60% of all invasive pneumococcal isolates. Serotype 3 was the dominant serotype, with the highest prevalence in patients ≥65 years of age. Penicillin susceptibility, increased exposure was 18.6%, mostly associated with serotypes 14 and 19A. Resistance to penicillin was low (<1%). Macrolide resistance was 23%, mostly associated with serotypes 14, 19A, and 19F. The coverage with 13-valent conjugate vaccine (PCV13) and 23-valent polysaccharide vaccine (PPV23) was 80.2% and 93.6%, respectively.

**Conclusions:**

The incidence of invasive pneumococcal disease in adults is highest in patients ≥65 years of age. Penicillin susceptibility, increased exposure and macrolide resistance were mostly associated with serotypes 14 and 19A. PCV13 and PPV23 provide very high serotype coverage. Future studies should evaluate the effects of the 10-valent vaccine, introduced in the Croatian National Immunization Program in June 2019, on serotype distribution and antibiotic resistance rates.

*Streptococcus pneumoniae* is among the most concerning human pathogens, with high morbidity and mortality rates worldwide. Pneumococcal infections range from non-invasive mucosal diseases (including acute otitis media, acute sinusitis, and pneumonia) to invasive, life-threatening infections (such as meningitis, sepsis, and bacteremic pneumonia) (1). Invasive pneumococcal disease (IPD) mostly affects children younger than 5 years and patients ≥65 years old (2). Every year, 500 000 children under 5 years of age die of IPD (3). Both morbidity and mortality rates are higher in developing countries (3). Community-acquired pneumonia is the most common pneumococcal disease worldwide, being responsible for more than 1.5 million of deaths annually. A significant fraction of these deaths are caused by *Streptococcus pneumoniae* (4,5).

Antimicrobial resistance of *Streptococcus pneumoniae* is a growing global health problem, mostly affecting penicillin and macrolides. The patterns of antimicrobial susceptibility differ among serotypes and geographic regions (6). Penicillin resistance has emerged within a few decades after penicillin introduction and has spread worldwide (7). The prevalence of antibiotic-resistant *Streptococcus pneumoniae* has been increasing (8,9). In Europe, the resistance rate in France, Spain, and Eastern European countries is concerning (10). Worldwide, some regions, such as South Africa, have antibiotic non-susceptibility rates of up to 79% (11). However, in the past several years some countries have reported decreased resistance rates (12-15).

Macrolide resistance is commonly present among invasive and non-invasive *Streptococcus pneumoniae* isolates. The main mechanisms are drug efflux system encoded by *mef* genes (M phenotype) and target modification mainly due to *erm*B genes, (MLS_B_ phenotype) (16,17).

The new fluoroquinolones or respiratory quinolones (levofloxacin, gatifloxacin, and moxifloxacin) have enhanced *in vitro* activity against *Streptococcus pneumoniae* and are used to treat respiratory tract infections in adults. Increasing resistance to fluoroquinolones has been reported in Asia and Africa (18,19). In addition, ineffectiveness of fluoroquinolones in the treatment of pneumococcal infections is associated with acquired resistance of *Streptococcus pneumoniae* to this group of antibiotics (20,21).

Increased resistance of *Streptococcus pneumoniae* to routinely used antibiotics warrants pneumococcal vaccine introduction as a tool for IPD prevention. In Europe, two pneumococcal vaccines are registered for use in adults: a 13-valent pneumococcal conjugate vaccine (PCV13, including serotypes 4, 6B, 9V, 14, 18C, 19F, 23F, 1, 5, 7F, 3, 6A, and 19A) and a 23-valent pneumococcal polysaccharide vaccine (PPV23, including PCV13 serotypes plus 1, 2, 5, 8, 9N, 10A, 11A, 12F, 15B, 17F, 20, 22F, and 33F) (22,23). In June 2019, a 10-valent pneumococcal conjugate vaccine (PCV10) was introduced in the Croatian National Immunization Program (NIP) for children only (scheme: 8 weeks – 16 weeks – 12 months) (24). In January 2021, the Croatian Public Health Institute revised its recommendations for pneumococcal vaccination of adults. Immunocompetent adults are now advised to be vaccinated with PPV23 only, while immunocompromised and asplenic patients are recommended to receive both vaccines, starting with PCV13 as the first dose (25). The aim of this study was to analyze the serotype distribution and antibiotic resistance of invasive *Streptococcus pneumoniae* isolates before the introduction of PCV10 in the childhood vaccination schedule, together with the coverage of currently available vaccines (PCV13 and PPV23). This study is the first and the most comprehensive so far in Croatia, analyzing invasive pneumococcal isolates collected during 15 consecutive years. These data will help us assess the impact of different vaccines in the IPD prevention among adults, especially those ≥65 years old.

## Material and methods

Invasive *Streptococcus pneumoniae* strains from entire Croatia are collected through a network of microbiological laboratories, coordinated by the Croatian Committee for Antimicrobial Resistance Surveillance, with country coverage >95%. The collection of invasive strains was introduced by the European Antimicrobial Resistance Surveillance System and is conducted within the frame of the European Antimicrobial Resistance Surveillance Network (EARS-Net). The laboratory support to the national antibiotic resistance surveillance, including the resistance surveillance of *Streptococcus pneumoniae*, is provided by the National Reference Center for Antimicrobial Resistance Surveillance (RCARS) at the University Hospital for Infectious Diseases in Zagreb. In this study, we analyzed the strains continuously collected from patients ≥18 years old in the period 2005-2019. The Croatian NIP does not include mandatory pneumococcal vaccination of adults. For this reason, we assessed the epidemiological parameters and vaccine coverage against IPD among Croatian adults. *Streptococcus pneumoniae* strains from primarily sterile samples (blood and cerebrospinal fluid, CSF) isolated in all microbiological laboratories in Croatia were delivered to the RCARS in a transport medium (Stuart or Aimes, Copan Diagnostics Inc., Corona, CA, USA). In RCARS, the isolates were subcultured to Columbia blood agar (Oxoid, USA), incubated at 37 °C in an atmosphere with 5% CO_2,_ 18-24 h, and stored in Schaedler-glycerol broth at -80 °C. The identification of *Streptococcus pneumoniae* strains was confirmed by Gram staining, the presence of α-hemolysis on blood agar, positive test with optochin disk, and positive bile solubility test (24-27). Antimicrobial susceptibility was tested by disk diffusion. In strains with reduced susceptibility to penicillin (as detected by oxacillin disk), minimum inhibitory concentration (MIC) was determined by using the gradient test (E-test, Biomerieux, Marcy-l'Étoile, France) according to the manufacturer`s recommendation. Until 2011, susceptibility testing was performed by following the recommendations of the European Committee for Antimicrobial Susceptibility Testing (28). *Streptococcus pneumoniae* strains were serotyped with latex agglutination method or Quellung reaction (29), as recommended by the manufacturer (Statens Serum Institute, Copenhagen, Denmark) (30). Macrolide resistance genes, *ermB* and *mefA,* were detected by polymerase chain reaction in erythromycin-resistant strains (31,32).

### Statistical analysis

Categorical variables are presented as medians with ranges, and interval variables are reported as means with standard deviations. The incidence rates were reported with Wald's 95% confidence intervals obtained by using R statistical software. IPD cases were calculated by using monthly data and a rolling window of twelve months. The differences in the prevalence of MLS_B_ phenotype compared with M phenotype were tested by using the Fischer's exact test with 2 × 2 tables.

## Results

**Figure 1:**
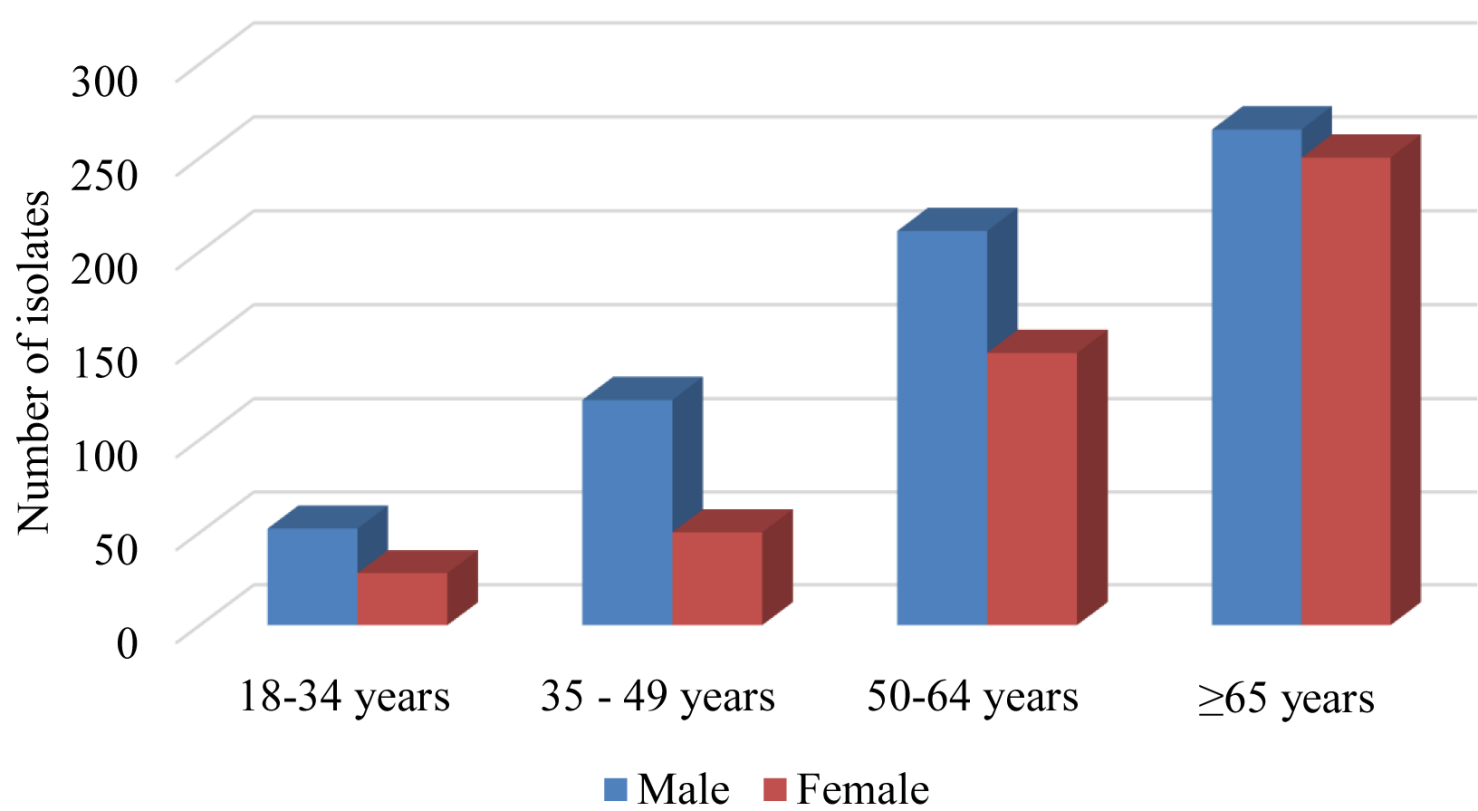
Distribution of confirmed invasive pneumococcal disease cases by age and sex.

During the fifteen-year study period, 1262 invasive isolates of *Streptococcus pneumoniae* were collected. A total of 1123 isolates were available for analysis, while 139 isolates were excluded due to insufficient clinical data or insufficient antibiotic susceptibility test results. All data were analyzed anonymously. The male-to-female ratio was 1.37:1. The mean age was 61 years (standard deviation ±16) and the median age was 62 years (range 18-97). The isolates were obtained from primary sterile sites: 975 isolates from blood (86.8%), 136 isolates from CSF (12.1%), and 12 isolates from both samples, taken from the same patient. Forty-six percent (515/1123) of all the isolates were from patients ≥65 years old (Figure 1). The incidence of confirmed IPD cases in adults 20-49 years old was 1.92/100 000 (95% confidence interval [CI] 0.45-2.34). In adults 50-64 years old, it was 2.68/100 000 (95% CI 0.39-3.07), while the highest incidence was among patients ≥65 years old, 4.45/100 000 (95% CI 0.79-5.24).

**Figure 2:**
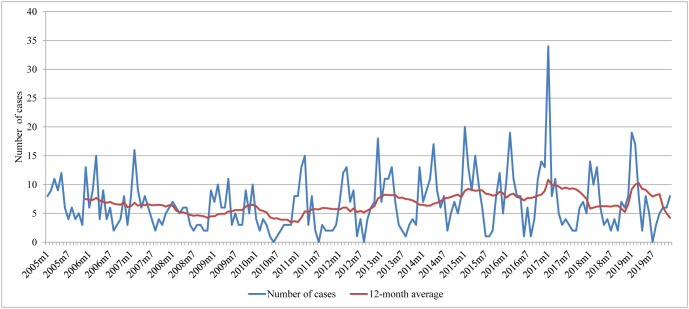
Distribution of confirmed invasive pneumococcal disease cases by month.

The number of IPD cases was lowest in summer, increased in autumn, and peaked in winter. After 2012, the 12-month average slightly increased (Figure 2). The most common clinical presentations of IPD were pneumonia (496 patients or 44.2%), sepsis (205 patients or 18.3%), meningitis (162 or 14.4%), febrile state (48 or 4%), and other clinical presentations (142 or 13%). Clinical presentation was unknown for 118 patients (11%). Information on underlying diseases that could have increased the risk of acquiring IPD was unavailable.

**Figure 3:**
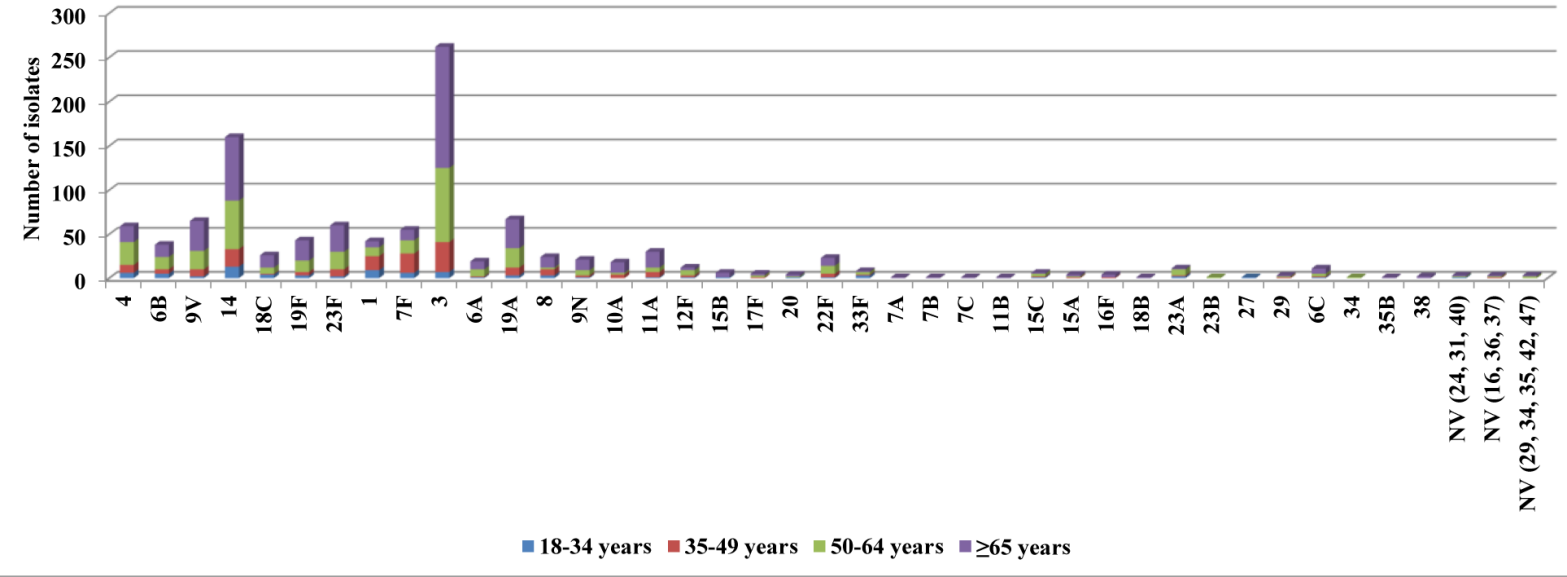
Serotype distribution of *Streptococcus pneumoniae* isolates by age group.

A total of 93% of all strains were serotyped. The remaining strains were not analyzed because they did not grow when subcultured from -80 °C. Among 38 invasive *Streptococcus pneumoniae* serotypes identified, the most prevalent serotypes were 3 (262 or 23.3%), 14 (160 or 14.2%), 19A (67 or 6%), 9V (65 or 5.8%), 7F (60 or 5.3%), and 23F (60 or 5.3%), comprising 60% of all invasive pneumococcal isolates. Serotype 3 had the highest prevalence in all age groups, peaking among patients ≥65 years old (137/515 or 26.6%) (Figure 3).

Among 496 isolates causing pneumonia, the most frequent serotypes were 3 (n = 127), 14 (n = 76), 7F (n = 36), 4 (n = 33), 19A (n = 31), and 23F (n = 30). Among 205 isolates causing sepsis, the most frequent serotypes were 3 (n = 47), 14 (n = 30), and 7F (n = 15). Serotype 3 was also the predominant serotype (n = 31) among 162 meningitis isolates, followed by serotypes 14 and 19F (n = 19 for each).

The coverage of PCV13 and PPV23 was 80.2% (901/1123) and 93.6% (1052/1123), respectively. In patients ≥65 years old, PCV13 and PPV23 vaccine coverage was 79.2% (408/515) and 93.9% (484/515), respectively. PCV13 and PPV23 coverage of isolates causing pneumonia, as the most common presentation of IPD in adults, was 85% (422/496) and 96.4% (478/496), respectively. PCV13 and PPV23 coverage of isolates causing meningitis, as the most severe presentation of IPD, was 77% and 90%, respectively.

**Figure 4:**
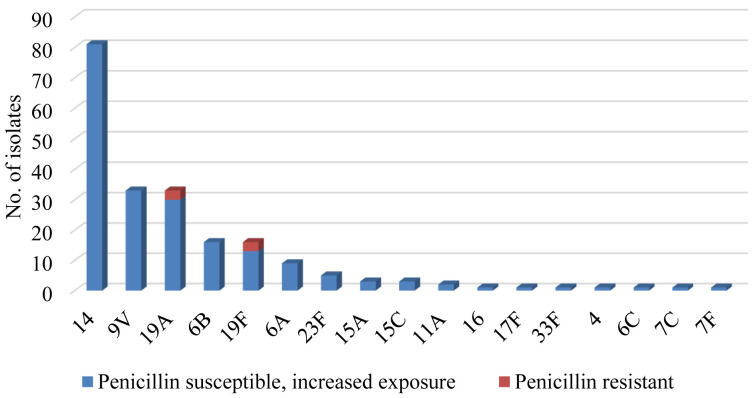
Serotype distribution of penicillin-resistant and susceptible, increased exposure *Streptococcus pneumoniae* isolates.

Penicillin susceptibility was tested in 99% of all isolates. Isolates with penicillin susceptibility, increased exposure (18.6%, 206/1108) mostly belonged to serotypes 14 (81/206, 39.3%), 9V (37/206, 18%), and 19A (30/206, 14.6%). Resistance to penicillin was low – less than 1% in the overall sample (Figure 4). Resistance to penicillin (MIC>2 mg/L) was detected in 6 isolates only, 3 isolates were serotype 19A and 3 were serotype 19F.

Antimicrobial susceptibility to third-generation cephalosporin, namely ceftriaxone, was tested in 98.5% of isolates (1107/1123). Susceptibility was 98.2%, while susceptibility, increased exposure was less than 2% (20/1107). Resistance to ceftriaxone was detected in 2 isolates only, which belonged to serotypes 19A and 19F.

**Figure 5:**
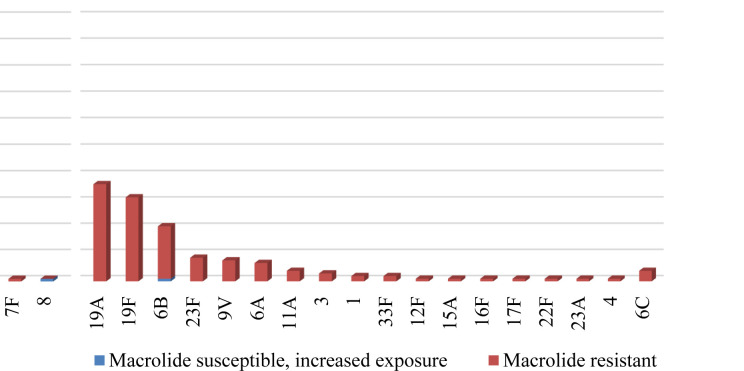
Serotype distribution of macrolide-resistant and susceptible, increased exposure *Streptococcus pneumoniae* isolates.

Macrolide susceptibility was tested in 93.2% of isolates. Resistance was 23% (238/1033), mostly associated with serotypes 14 (100 isolates/42%), 19A (37 isolates/15.5%), and 19F (32 isolates/13.4%) (Figure 5). The presence of macrolide resistance genes was tested in 202 out of 241 erythromycin-resistant isolates (85.1%). Resistance genes were detected in 173/202 (85.6%) isolates: 104 isolates (51.5%) were e*rmB* positive, 64 isolates (31.7%) were *mefA* positive, and 5 isolates (2.5%) were positive for both genes. A total of 29 strains (14.3%) were negative for both genes. MLS_B_ phenotype was significantly more prevalent than M phenotype (*P* < 0.05).

Resistance to fluoroquinolones was detected in 4 isolates, belonging to serotypes 23F, 22F, 9V, and 19F.

The coverage of PCV13 and PPV23 vaccines for penicillin-susceptible, increased exposure and resistant isolates was 93.8% (199/212) and 95.7% (203/212), respectively. The coverage for macrolide-resistant strains was 92.4% (220/238) and 96.2% (229/238), respectively.

## Discussion

In this study, the incidence of invasive pneumococcal disease in adults was highest in patients ≥65 years of age. Penicillin susceptibility, increased exposure and macrolide resistance were mostly associated with serotypes 14 and 19A. PCV13 and PPV23 provided very high serotype coverage. The seasonal distribution of IPD in adults in Croatia was similar to that in many other European countries. The distribution followed the pattern typical for most respiratory diseases, with the greatest number of infections occurring during the winter months. Furthermore, a male predominance was noticed (6).

More than 100 different serotypes of *Streptococcus pneumoniae* have been identified but most of IPD are still caused by a small number of serotypes. Due to the different serotype prevalence in different countries, it is crucial to identify the most common serotypes and serotypes with acquired resistance to antibiotics in each country (6). Differences in serotype prevalence among countries are linked to differences in vaccines used and times of vaccine introduction (7,10,33-36). In addition, the prevalence of different serotypes oscillates naturally, even without conjugate vaccines pressure (37).

In our study, six most common serotypes (3, 14, 19A, 9V, 7F, and 23F), included in both PCV13 and PPV23, comprised 60% of all invasive pneumococcal isolates. In many countries, the introduction of conjugate vaccines was followed by a significant decrease in IPD caused by serotypes included in the vaccine and a partial replacement of these serotypes by non-vaccine serotypes (6,38-40).

Currently, the prevalence of non-vaccine serotypes 8, 9N, 15A, and 23B is increasing in Australia, USA, France, and Norway (6,41). Furthermore, in countries that introduced conjugate vaccines, the predominant serotypes were not present, as was seen with serotype 19A after the introduction of a 7-valent conjugate vaccine and serotype 14 in the pre-vaccination period (41).

In our study, serotype 3 was by far the most prevalent serotype in all age groups, with the highest prevalence among patients ≥65 years old. Due to immunosenescence and co-morbidities, these patients are at higher risk for developing IPD and are therefore recommended to receive pneumococcal vaccination (42,43). In some European countries, serotype 3 remained high in PCV13 post-vaccination period, which can be explained by low vaccine immunogenicity for that serotype or high carriage rate among patients ≥65 years old (44).

In this study, the coverage of the currently available vaccines among adult population was 81.1% for 13-valent PCV and 94.5% for 23-valent PPS. Regarding the clinical presentations, we observed no important differences from other EU countries. Pneumonia was the most common cause of IPD in adults – accounting for 44.2% of all IPD episodes (45). The most prevalent serotypes causing pneumonia were serotypes 3 and 14, both included in the vaccines registered for adults.

These findings highlight the importance of introducing pneumococcal vaccine for adults in the Croatian NIP. Since 1987, only PPV23 has been available for adult immunization. In 2011, European countries and the USA additionally approved PCV13 for use in adults older than 65 years. In 2015, PCV13 use was extended for all adults ≥18 years. In Croatia, PPV23 was registered in 2014 for adults and children ≥2 years. Registration dates for PCV13 were the same for all EU countries, (2005 – children 6 months-5 years, 2011 – adults ≥50 years, 2012 – children 6-17 years, and 2013 – adults 18-49 years). Both vaccines have a high coverage of invasive pneumococcal isolates combined with a high coverage of the resistant isolates. Pneumococcal conjugate vaccines have been associated with a reduced antibiotic consumption, which, in the long-term, contributes to lower antimicrobial selection pressure (46,47).

Penicillin-susceptible, increased-exposure and penicillin-resistant pneumococcal isolates were detected in 18.6% and <1% of all isolates, respectively. Penicillin remains a drug of choice for non-meningitis pneumococcal infections caused by penicillin-susceptible, increased-exposure isolates, while meningitis pneumococcal infections require the treatment with a fully susceptible antibiotic (48).

Increased prevalence of penicillin non-wild type pneumococcal isolates is a result of beta-lactams overuse (49). Based on the surveillance data of antibiotic consumption in Croatia in 2019, co-amoxiclav was the most prescribed antibiotic in outpatient settings, followed by amoxicillin and cefuxime-axetil (50). The introduction of the higher-valent vaccines significantly decreased the number of multidrug-resistant pneumococcal strains, including penicillin non-wild type and multidrug resistant strains (reduction by 7.4% and 6.9%, respectively). This is due to the decrease in the number of IPD cases caused by vaccine serotypes accompanied by a lower antibiotic consumption (51).

Although pneumococcal isolates in this study showed acquired resistance to penicillin, they did not show resistance to other beta-lactams, such as third-generation cephalosporins, namely ceftriaxone. Ceftriaxone-susceptible, increased-exposure pneumococcal isolates were detected in <2% of all isolates, which makes this antibiotic the first choice for empirical therapy of pneumococcal meningitis. Similarly, in many European countries, amoxicillin is the first drug of choice in treatment guidelines for community-acquired pneumonia due to its good *in vitro* activity and clinical effectiveness (52). Amoxicillin susceptibility of invasive and non-invasive *Streptococcus pneumoniae* isolates in Croatia in 2019 was 86% (50).

In our study, macrolide resistance was 23.5%, mainly due to MLS_B_-phenotype (51.5%). In other European countries and the USA, M-phenotype was the predominant resistance mechanism (53,54). We observed only five isolates with both resistance genes detected. Some of our pneumococcal isolates (29 isolates) were negative to both genes, which indicates the possibility of some other resistance mechanisms.

Recently, macrolide resistance has been connected to mutations in the 23S rRNA or modification of the ribosomal proteins L4 and L22, but additional research into this issue is warranted (51,55). Antibiotic overuse has been confirmed as the major driver of antibiotic resistance (49). EARS-Net surveillance data for Croatia during the observed period showed varying macrolide resistance rates of invasive pneumococcal isolates, ranging from 8% in 2007 to 37% in 2017. In the last two years, resistance rates appear to be decreasing, being 30% in 2019. The decrease could have resulted from a lower macrolide use in the empirical therapy of suspected pneumococcal infections due to a continuous high resistance rate to macrolides. Macrolide resistance of both invasive and non-invasive pneumococcal isolates was lowest in 2005 (28%) and highest in 2008 (40%). Since 2011, it has been slowly decreasing, reaching 31% in 2019 (50). Goossens et al (56) analyzed macrolide use and macrolide resistance in 26 countries, showing that the outpatient macrolide use correlated well with macrolide resistance – with Greece being the country with the highest use.

In our study, fluoroquinolone resistance remained very low (<1%). Increased fluoroquinolone use in the outpatient setting in Croatia (8% in 2019) did not affect the resistance rate of invasive isolates (55,57). In many countries, low level of fluoroquinolone resistance, below 2%, remained stable despite the introduction of PCVs and consequent serotype replacement (58-60).

The limitation of this study was that the analysis did not include IPD cases confirmed by molecular diagnostics only. Regardless of this limitation, the results of our study represent an important contribution to the understanding of the epidemiology and burden of IPD among the adult population in Croatia before the introduction of PCV10. Our data on serotype distribution and antimicrobial resistance among invasive pneumococcal isolates, and potential vaccine coverage with available vaccines, could be used to inform the strategies for IPD prevention in Croatian adults.
